# Silymarin reduces retinal microvascular damage in streptozotocin-induced diabetic rats

**DOI:** 10.1038/s41598-022-20297-2

**Published:** 2022-09-23

**Authors:** Rahman Karimi, Ali Bakhshi, Parisa Dayati, Omid Abazari, Maryamsadat Shahidi, Mohamadreza Savaee, Ehsan Kafi, Mehdi Rahmanian, Seyed Morteza Naghib

**Affiliations:** 1 Department of Cell & Molecular Biology, Faculty of Biological Sciences, Kharazmi University, Tehran, Iran; 2 Department of Clinical Biochemistry, School of Medicine, Shahid Sadoughi University of Medical Sciences and Health Services, Yazd, Iran; 3 Department of Clinical Biochemistry, Faculty of Medical Sciences, Tarbiat Modares University, Tehran, Iran; 4 Biomaterials and Tissue Engineering Department, Breast Cancer Research Center, Motamed Cancer Institute, ACECR, Tehran, 1517964311 Iran; 5 Nanotechnology Department, School of Advanced Technologies, Iran University of Science and Technology (IUST), Tehran, Iran

**Keywords:** Drug regulation, Drug safety, Drug screening

## Abstract

Diabetic retinopathy is a severe microvascular problem in diabetes mellitus. Silymarin is a flavonoid compound, and according to previous studies, it is a bioactive compound with potent antioxidant and anti-inflammatory properties. This investigation aims to peruse the impact of silymarin against diabetic retinopathy in streptozotocin (STZ)-provoked rats. Thirty-two adult male Wistar rats were randomly allocated into the control group, STZ group, STZ + silymarin (50 mg/kg), and STZ + silymarin (100 mg/kg). STZ rats received silymarin every day until 2 months after diabetes induction. The serum and retinal tissues were collected 2 months after silymarin treatment to determine biochemical and molecular analyses. Silymarin markedly lowered the serum glucose concentration in diabetic rats. Silymarin reduced the increased levels of advanced glycosylated end products (AGEs), the receptors for AGEs (RAGE), and reactive oxygen species (ROS) in diabetic rats. Silymarin also attenuated the phosphorylation of p38 MAP kinase and nuclear factor (NF)-κB p65 and diminished diabetes-induced overexpression of inflammatory cytokines, vascular endothelial growth factor (VEGF), adhesion molecules, and extracellular matrix proteins in STZ rats. Our data suggested that silymarin has protective effects against diabetic retinopathy, which might be related to the inhibition of the AGEs/RAGE axis and its antioxidant and anti-inflammatory activities.

## Introduction

Diabetic retinopathy is considered one of the leading causes of irreversible visual loss in diabetes mellitus^1^. Globally, about one-third of patients with diabetes mellitus have symptoms of diabetic retinopathy^2^. Therefore, there is an immediate demand to recognize impressive strategies for preventing and treating diabetic retinopathy.

Clinically, this complication is divided into non-proliferative and proliferative stages. The non-proliferative stage is characterized by intraretinal microvascular abnormalities, consisting of increased permeability of retinal vasculature, macular edema, increased thickness of capillary basement membrane, and capillary occlusion. The abnormal formation of new blood vessels on the retina's inner surface and retinal fibrosis can be detected during the proliferative stage as the advanced stage of diabetic retinopathy. These alterations caused severe vision problems when the new abnormal vessels grew into the vitreous cavity, leading to vitreous hemorrhage^3,4^. The visual impairments may be prevented early in the disease, but it becomes more difficult to manage in the advanced stage^1^.

However, many signaling pathways may be involved in retinal microvascular damage. In diabetes mellitus, hyperglycemic conditions lead to the increased generation of advanced glycation end products (AGEs), which are considered one of the primary contributors to the development and progression of diabetic retinopathy^5^. AGEs are the end product of a non-enzymatic glycation reaction between proteins and reducing sugars, the Millard reaction^6^. The formation of AGEs in retinal vasculature led to the disruption of vessel integrity, resulting in vascular obstruction, increased vascular leakage, retinal ischemia, and neovascularization^7^. Increasing evidence demonstrated that AGEs and the receptor for AGEs (RAGE) were detected in the serum and retinal vasculature of patients with diabetic retinopathy. At the molecular level, AGEs bind to RAGE and trigger a series of cellular events, including excessive reactive oxygen species (ROS) generation, which upregulates the status of nuclear factor kappa-B (NF-κB) proinflammatory factors^8^. In the retina, proinflammatory cytokines provoke the adhesion of leukocytes to the vascular endothelium, which leads to the permeability of the blood-retinal barrier (BRB). Besides, AGEs also upregulate vascular endothelial growth factor (VEGF), which plays a crucial role in leukocyte-mediated BRB breakdown and neovascularization^7,9^. Regarding the central part of the AGE-RAGE pathway in the pathophysiology of diabetic retinopathy, the blockade of this axis has been suggested as a novel therapeutic option for improving microvascular damages in diabetic retinopathy.

In traditional medicine, many medicinal plants with hypoglycemic properties have been introduced for treating diabetes mellitus^10^. Silymarin, a flavonoid compound isolated from Silybum marianum (Milk thistle), has been used as a herbal remedy in traditional medicine for various diseases^11^. Generally, it has been utilized to treat liver problems^12^. In addition, it is revealed that this flavonoid compound has a precious antidiabetic property due to its antioxidant and anti-inflammatory properties^13^. It has been shown that silymarin could effectively improve glucose and lipid profiles, scavenge ROS, and inhibit the production of proinflammatory cytokines in diabetic rats^14–16^. Thus, silymarin may be useful for controlling diabetic complications^15^. Previously, the effect of silymarin has been reported on the vascular leakage of human retinal endothelial cells^17^. However, the possible protective effects of silymarin on retinal tissues in a diabetic animal model are yet to be appraised.

Following this assumption, the current research was designed to determine the favorable impact of silymarin on retina damage in diabetes. Streptozotocin (STZ) is an antibiotic derived from Streptomyces achromogenes that causes irreversible destruction of pancreatic islet β-cell and is extensively used to induce diabetes mellitus in experimental animals. Rats are sensitive to STZ-induced pancreatic β-cell destruction and are a suitable animal model for experimental studies in diabetes^18^. Therefore, STZ was employed to induce diabetic rats in this study. Subsequently, silymarin's possible molecular protective mechanism is detected by AGEs, RAGE, ROS levels, p38 MAP kinase, and NF-κB pp65 phosphorylation and inflammatory parameters, adhesion molecules, and extracellular matrix proteins in retinal tissue.

## Materials and methods

### Ethical statement

The procedures related to the maintenance and use of laboratory animals were approved by the Animal Ethics Committee of Shahid Sadoughi University of Medical Sciences (approval number: IR.SSU.MEDICINE.REC.1400.358) and conducted by the National Institutes of Health (NIH) guidelines (No 80–23, revised 1996). All experimental procedures were also performed and reported according to the recommendations in ARRIVE guidelines. All efforts such as sacrificing and environmental conditions were made to minimize suffering and distress. This article only has animal subjects performed by the authors.

### Animals

A total of 32 adult healthy male Wistar rats (weighing 220–250 g and 2 months of age) were included in this experimental animal research. All rats were provided from the animal lab of Shahid Sadoughi University of Medical Sciences (Yazd, Iran) and housed (n = 4 rats/cage) under controlled conditions (24 °C ± 2 °C, 60–80% humidity, and 12 h light/dark cycle) for 1-week adaptation. All rats had free access to both standard food and water. The research team monitored daily animal activity, food and water intake, and environmental conditions. The sample size was computed by the G* Power software (Version 3.9.1.4), considering an alpha error of 0.05 and power of 0.8. A total of 8 rats per group were calculated. The animals were excluded if diabetes was not induced in diabetic groups or the animals died.

The cages were chased randomly from the pool of all cages. Each rat was given a random number to ensure that all rats were monitored and treated similarly. So, those who treated rats were not aware of allocation groups. Before data analysis, the grouped data were coded so that the control and treatment groups could not be recognized until the studies were completed. Also, an investigator analyzed all results with no information about codes related to treatment and control groups.

### Treatment protocols

All protocols were designed and prepared before the study. The animals were randomized using list randomizer (https://www.random.org/lists) into four groups (n = 8 rats/ each group): (1) control group, (2) diabetic group, (3) diabetes + silymarin (50 mg/kg), and (4) diabetes + silymarin (100 mg/kg).

The animals of control and diabetic groups orally received vehicle (distilled water). In treatment groups, diabetic rats orally received 50 or 100 mg/kg of silymarin^19,20^ once daily for 8 weeks^21,22^. Silymarin (S0292) was purchased from Sigma Company (USA) and prepared in a vehicle with 1.5 ml/kg distilled water. The doses were selected based on the prior studies that showed that silymarin improved diabetes in STZ-diabetic rats^20^. Due to none of the animals dying, 32 rats (n = 8/each group) were included in all further analyses. The study monitored and recorded the body weight and blood glucose level.

### Diabetes induction

To induce type 1 diabetes in the rats assigned to groups 2, 3, and 4, the animals received an intravenous injection of STZ (60 mg/kg; Sigma, USA) prepared in sodium citrate buffer (10 mM, pH 4.5)^19,23,24^. The animals in group 1 received the same volume of sodium citrate buffer from the same person. Tail vein blood glucose level was monitored 3 days after STZ injection using a glucose meter (Roche Diagnostics K.K., Tokyo, Japan). If rats had blood glucose ≥ 300 mg/dL, they were considered diabetes rats^25^. As expected, all animals in groups 2, 3, and 4 were successfully diabetic.

### Measurement of body weight

After the experimental period, body weight was determined to the nearest 0.1 kg on a digital balance (model 707; Seca, Germany).

### Sample collection

After 8 weeks of silymarin treatment, all fasted overnight rats were euthanized following anaesthetization with a single intraperitoneal injection of 100 mg/kg ketamine and 10 mg/kg xylazine mixture. The whole blood samples were taken via heart puncture of anesthetized rats. The blood samples were centrifuged immediately (2000*g*,10 min, 4 °C), then their serums were separated and kept at − 80 °C for subsequent biochemical measurements. Subsequently, the rat’s eyes were quickly removed, and retinal tissues were dissected from the cornea, lens, and choroidal tissues using sterile forceps and scissors. The retinal tissues were homogenized manually with a glass homogenizer and preserved at − 80 °C until the subsequent molecular analyzes. The animals were sacrificed at 8 am to lessen the circadian effect.

### Measurement of serum glucose

Serum glucose level was detected using a colorimetric assay kit from Pars Azmoon (Iran, Tehran), following the protocols provided by the manufacturer.

### Retinal AGEs determination

The retinal tissues were homogenized and lysed using an ice-cold RIPA buffer containing protease inhibitor cocktail. The obtained homogenates were centrifuged at 10,000*g* for 20 min in 4 °C^26^. The AGEs concentrations in the supernatant fraction of retinal tissue homogenates were quantified using Rat AGEs ELISA kit concurred from MyBioSource, Inc. (USA). The sensitivity and detection range for Rat AGEs ELISA kit was 500–7.8 ng/ml and 0.8 ng/ml, respectively.

### Retinal ROS detection

2′-7′-dichlorofluorescein-diacetate (DCFH-DA, Sigma Aldrich, USA) probe was applied to monitor ROS levels in retinal tissue homogenate^27^. DCFH-DA is a nonfluorescent dye that ROS can oxidize to form dichlorofluorescein (DCF), a fluorescent molecule. In brief, rat retinal tissue samples were homogenized in PBS using a glass homogenizer in the presence of protease inhibitors. Subsequently, samples were suspended in 200 μl of PBS (1×) and exposed to 5 μM DCFH-DA for 15 min at 37 °C in darkness. Fluorescence was measured at 488 excitations and 525 emission wavelengths, respectively^28^. The fluorescence intensity was reported based on the Arbitrary Fluorescence Units (AFU).

### Real-time PCR

Following the working procedure, total RNA isolation from rat retinal tissue was performed using a Trizol reagent (Bio Basic, Canada). A nanodrop spectrophotometer (Thermo Fisher Scientific, USA) was supplied to measure isolated RNA's quantity and quality. Then, the RNA integrity was evaluated on 1% agarose gel. Both 18 s and 28 s ribosomal RNA bands were observed without extra bands. Subsequently, 1 μg of total RNA per sample was exploited using a suitable kit for the reverse transcriptase reaction (Pars Tus, Iran). Finally, gene expression was determined by quantitative real-time PCR using specific primers (Takapouzist, Iran) and an appropriate SYBR Green qPCR master mix (Yekta Tajhiz company, Iran) on a 7500 Real-Time PCR System (Applied Biosystems). The primer sequences of target genes are presented in Table 1.

**Table 1:** The primer sequences of target genes.

Gene	Primers sequences
TNF-α	Forward: 5′-ACACCATGAGCACGGAAAGC-3′ Reverse: 5′-CCGCCACGAGCAGGAA-3′
IL-1β	Forward: 5′-AATGGACAGAACATAAGCCAACA-3′ Reverse: 5′-CCCAAGGCCACAGGGAT-3′
IL-6	Forward: 5′-GTTGCCTTCTTGGGACTGATG-3′ Reverse: 5′-ATACTGGTCTGTTGTGGGTGGT-3′
VEGF	Forward: 5′-CACTGGACCCTGGCTTTACT-3′ Reverse: 5′-TCAATTGGACGGCAATAGCT-3′
RAGE	Forward: 5′-AAGCCCCTGGTGCCTAATGAG-3′ Reverse: 5′-CACCAATTGGACCTCCT-CCA-3′
ICAM-1	Forward: 5′-CGGGTTTGGGCTTCTCC-3′ Reverse: 5′-GCCACTGCTCGTCCACATAG-3′
VCAM-1	Forward: 5′-ATCTTCGGAGCCTCAACGG-3′ Reverse: 5′-CCAATCTGAGCGAGCGTTT-3′
TGF-β	Forward: 5′-CAATTCCTGGCGTTACCTTG-3′ Reverse: 5′-AAAGCCCTGTATTCCGTCTC-3′
Collagen IV	Forward: CCATTCTCAGGACTTGGGTA Reverse: AAGGGCATGGTGCTGAACT
Fibronectin	Forward: CAGCCCCTGATTGGAGTC Reverse: TGGGTGACACCTGAGTGAAC
β-actin	Forward: 5′-TGTGATGGTGGGAATGGGTCAG-3′ Reverse: 5′-TTTGATGTCACGCACGATTTCC-3′

### Western blot analyses

Retinal tissues were collected and homogenized in 1 mL of ice-cold RIPA buffer containing protease inhibitor cocktails. Then, the lysates were centrifuged for 10 min at 14,000×*g* at 4 °C^26^. The supernatants were collected, and total protein amounts were quantified using a Bradford protein assay kit (DNAbiotech Co. IR. Iran)^29^. Subsequently, proteins (30 µg) were resolved on 12% sodium dodecyl sulfate–polyacrylamide gel electrophoresis (SDS-PAGE) and moved onto polyvinylidene difluoride (PVDF, Sigma Aldrich) membranes as a support material. After blocking with 5% non-fat dry milk blocking buffer (5 g powder per 100 ml of TBS plus 0.2% Tween 20), the membranes were exposed to the primary antibodies at 4 °C overnight anti-RAGE (1:1000; Cat. No. sc-5563; Santa Cruz, CA, USA), anti-NF-κB pp65 (1:1000; Cat. No. sc-166748; Santa Cruz, CA, USA), anti-pp38 (1:1000; Cat. No. sc-166182; Santa Cruz, CA, USA), and anti-GAPDH (1:10,000; Cat. No. ab181603; Abcam), followed by the secondary antibody (1:5000; Cat. No. ab6721, Abcam) for 1 h at 25 °C. Then, the blots were monitored using an enhanced chemiluminescence detection kit on a Bio-Rad imaging system (USA) and analyzed using Image J software.

### Data analysis

All statistical analyses were conducted using Graph-Pad Prism software version 8.0 for Windows (Graph-Pad Software Inc., San Diego, CA). Normality of all data was determined by Kolmogorov–Smirnov (K-S) test. All findings (n = 8 per group) are reported as mean ± standard deviation (SD). The differences between multiple groups were compared using a one-way analysis of variance (ANOVA), followed by Tukey’s post hoc test. P < 0.05 was set as a statistical significance.

## Results and discussion

**Figure 1:**
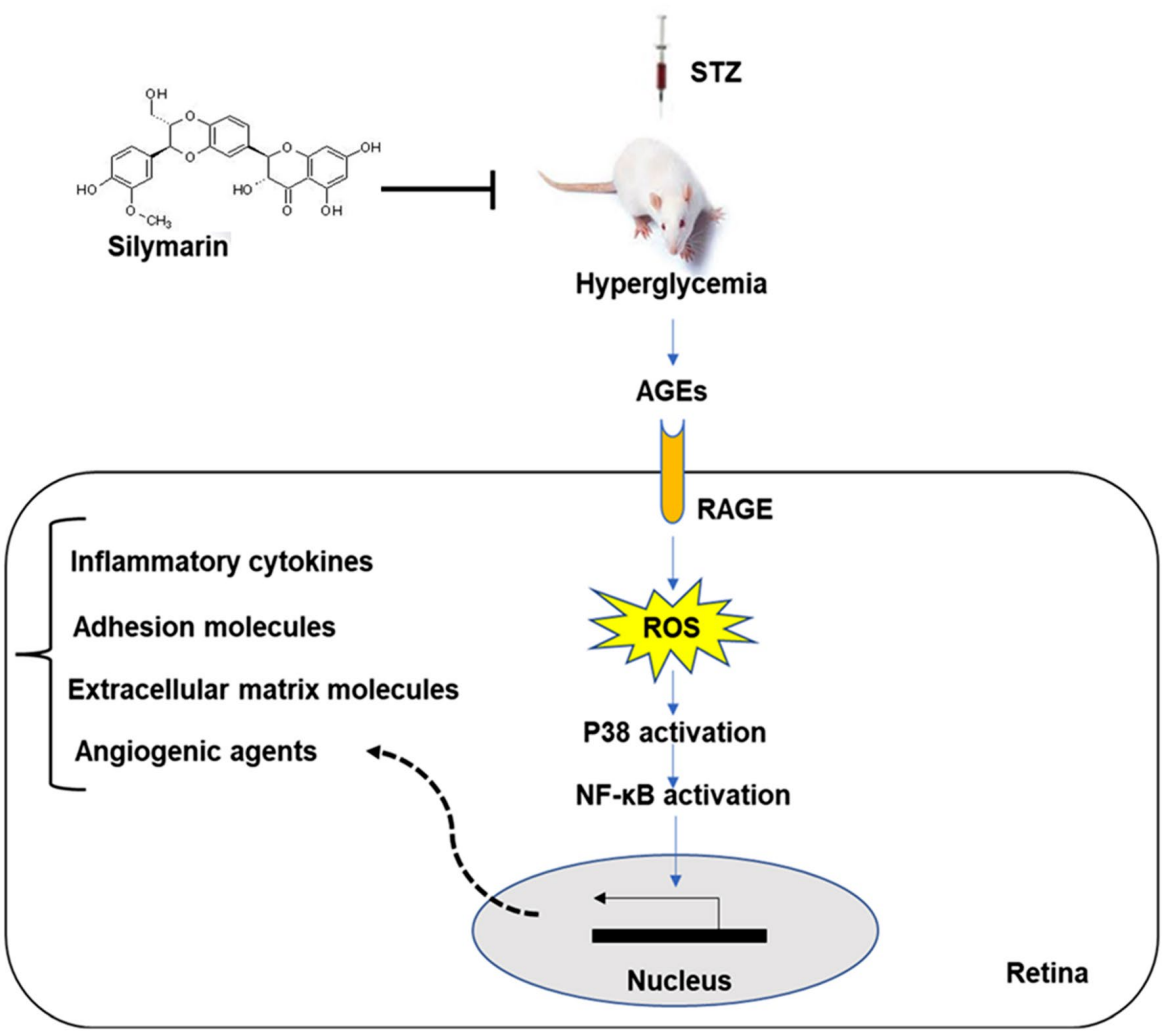
Schematic of the possible effect of silymarin on reducing retinal damage in STZ-induced diabetic rats.

This manuscript reported that silymarin administration substantially ameliorated diabetic retinopathy in a diabetic rat model through attenuation of AGEs, RAGE, and ROS levels as well as reduction of p38 MAP kinase and NF-κB pp65 phosphorylation and consequently inhibition of inflammatory parameters, adhesion molecules, and extracellular matrix proteins (Fig. 1).

Diabetes mellitus describes a group of metabolic disorders usually characterized by abnormal glucose homeostasis due to impaired insulin secretion, insulin function, or both^30^. Diabetic retinopathy is a severe microvascular complication of diabetes and affects about one-third of diabetic patients^4^. Despite great advancements in glycemic control through nutritional therapy and pharmacological interventions, managing diabetic complications such as visual impairments remains a significant challenge in clinical practice. Since conventional drug treatments for diabetic complications are not always satisfactory, interest in alternative and complementary therapies grows^31^.

Herbal remedies have been applied as a safe source of therapeutic compounds for treating many illnesses in traditional medicine^32,33^. It was found that many herbal remedies improved blood glucose levels and insulin resistance in diabetic patients^31,34^. Some protective mechanisms of silymarin against retinopathy have been suggested by recent in vivo and in vitro observations^20,22,35^. The mechanisms implicated in retinopathy include retinal neurodegeneration, damaged retinal vessel, and neuroinflammation^36,37^. Bartoli et al. showed that silymarin positively impacted diabetic retinopathy by preventing stress-induced premature senescence, preserving retina cells survival, and reducing the expression of pro-inflammatory as well as pro-oxidant factors^38^.

Additionally, Zhang et al. demonstrated that silybin, a major active compound of silymarin, prevented the development of obliterated retinal capillaries, decreased retinal vascular leukostasis, and retinal ICAM-1^39^. In another study, silybin prevented VEGF secretion, neovascularization, and retinal edema in rats^40^. Besides, silymarin caused a significant reduction in VEGF-induced permeability induced by diabetic conditions in human retinal endothelial cells^17^.

**Figure 2:**
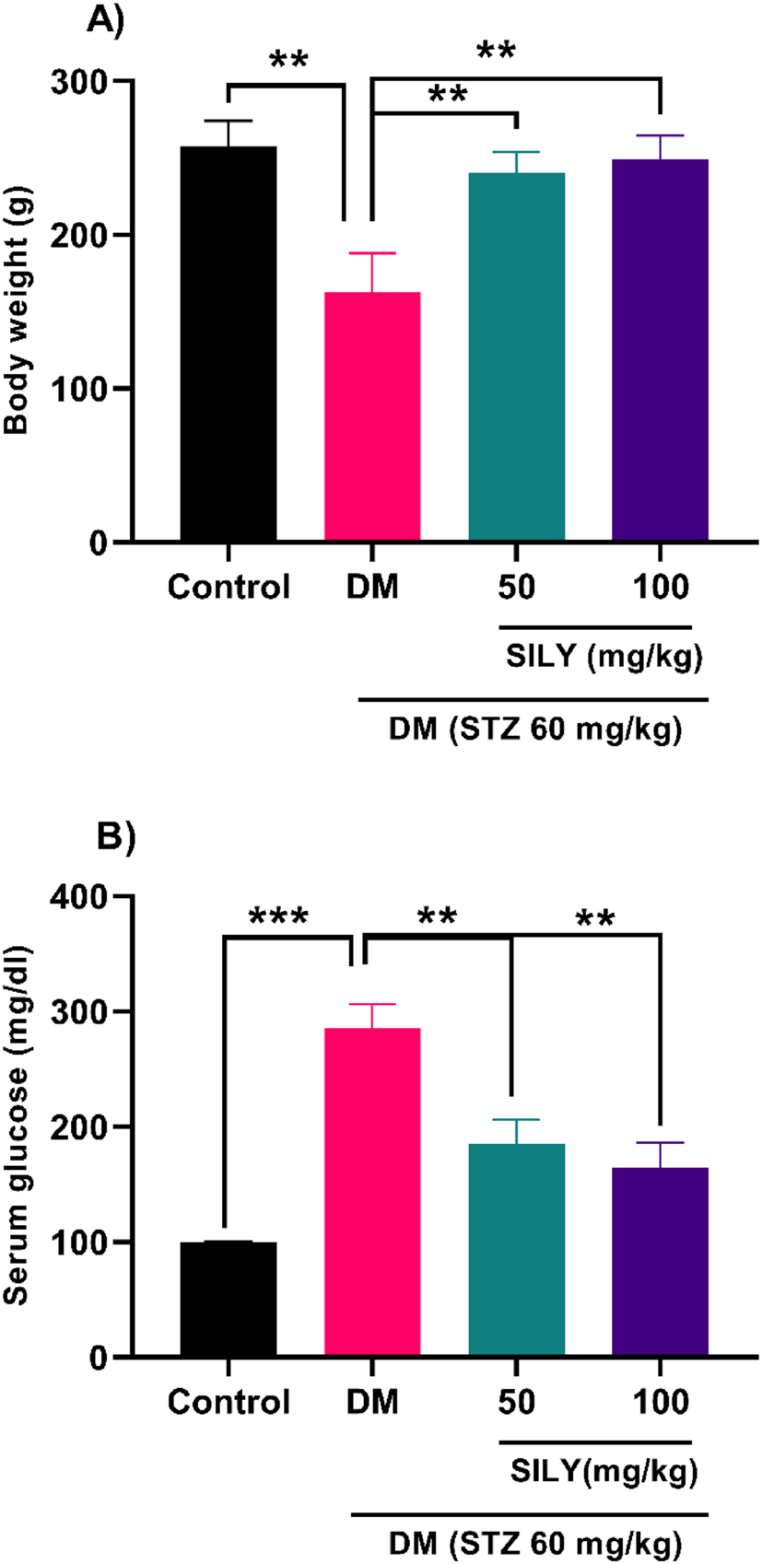
Effect of silymarin on (**A**) body weight and (**B**) serum glucose in diabetic rats. Data are reported as mean ± standard deviation (SD). The differences between groups were compared using one-way ANOVA and Tukey’s post hoc test. Eight animals in each group were utilized for this experiment. ** and *** showing *p* < 0.01 and *p* < 0.001, respectively. DM: diabetes mellitus; SILY: silymarin.

Considering that all impacts of silymarin against diabetic retinopathy have not been studied so far, the present study aimed at investigating the effects of silymarin on the AGE-RAGE axis and its downstream targets, specifically in a rat model. Because body weight is often altered in pathophysiological conditions like diabetes, we first assessed the effects of silymarin on body weight in STZ-provoked rats. At the end of the experimental period, an obvious weight loss was found in the diabetic group relative to the healthy group (Fig. 2A; P < 0.01). Our findings were in agreement with previous studies. In a prior study conducted by Eleazu and his co-workers, the bodyweight reduction in the STZ-induced diabetic rats was attributed to the degradation of structural proteins^41^. Kusari et al. reported that body weight loss in the STZ-induced diabetic rats could be associated with muscle breakdown due to hyperglycemia^42^. However, 8 weeks of silymarin treatment substantially raised the body weight in the diabetic groups receiving silymarin relative to the diabetic group (Fig. 2A; P < 0.01). This effect of silymarin may be due to its potential hypoglycemic effects^43^. As depicted in Fig. 2B, the serum glucose level was dramatically augmented in the diabetic group relative to the healthy group (P < 0.001). Elevated blood glucose levels can be due to dysfunction of β cells and insulin secretion following STZ-provoked diabetes^44^. On the other hand, 8 weeks of treatment of diabetic animals with silymarin appreciably lowered the serum levels of glucose (Fig. 2B; P < 0.01).

**Figure 3:**
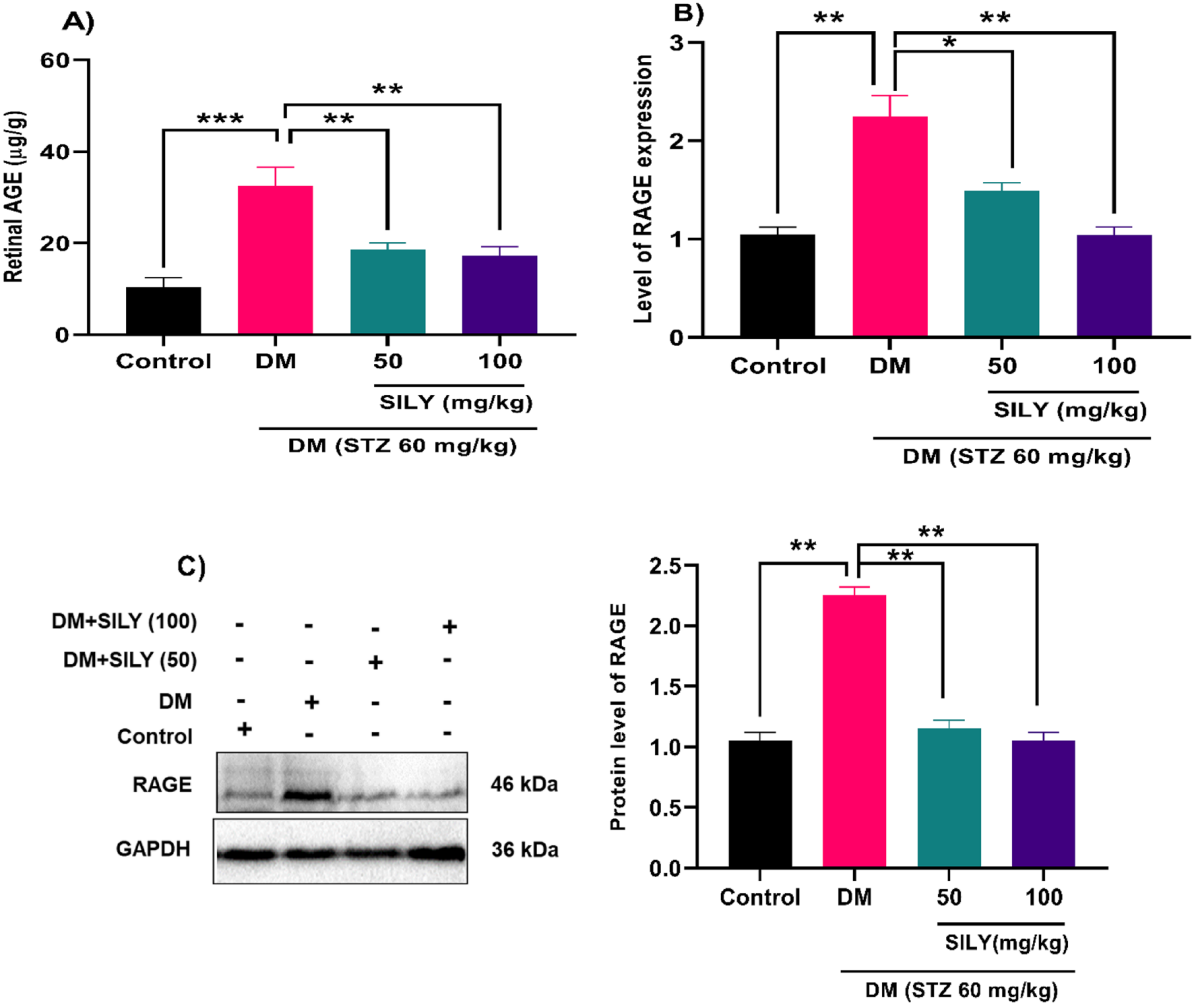
Silymarin attenuates (**A**) AGEs and (**B**,**C**) RAGE levels in the retina of diabetic rats. Q-RT-PCR and western blotting were applied to quantify mRNA and protein, respectively. Data are reported as mean ± SD. The differences between groups were compared using one-way ANOVA and Tukey’s post hoc test. Eight animals in each group were utilized for this experiment. The original blot was presented in Supplementary Fig. S1. *, ** and *** showing *p* < 0.05, *p* < 0.01, and *p* < 0.001, respectively. DM: diabetes mellitus; SILY: silymarin; AGE: advanced glycation end products; RAGE: Receptor for Advanced glycation end products.

One metabolic consequence of consistent hyperglycemia is the accelerated production of AGEs, the deposition of which in retinal tissue is closely connected with the pathogenesis of diabetic retinopathy^5^. Several studies have revealed that AGEs in serum and retinal vasculature of patients with diabetic retinopathy were higher than that of normal individuals, indicating the association between abnormal levels of AGEs and the development of diabetic retinopathy^45,46^. Increasing evidence has disclosed that the accumulation of AGEs and their receptor, RAGE, altered the expression of multiple downstream targets changes as downstream target proteins, including NF-κB, VEGF, and extracellular matrix proteins in retinal microvascular endothelial cells^5^. Additionally, it was proved that AGEs could exacerbate the progressive death of retinal neuronal cells^47^. Therefore, effective control of the AGE-RAGE axis effectively reverses diabetes-related problems in patients^48^. Previous studies demonstrated that phytochemical treatment potentially attenuated the overproduction of AGEs and RAGE in STZ-induced diabetic rats^26,49^. For instance, Tzeng et al. reported that Zerumbone administration (40 mg/kg) downregulated the increased levels of AGE and RAGE in the retinas of STZ-diabetic rats^26^. The findings of our study exhibited that the AGEs protein level was elevated in the retina of the diabetic group relative to the healthy group (Fig. 3A; P < 0.001), which were notably reduced after treatment with 50 and 100 mg/kg of silymarin (P < 0.01). According to q-RT-PCR and western blot analyses, there is a notable increase in RAGE mRNA and protein levels in the retina of the diabetic group relative to the healthy group (Fig. 3B,C; P < 0.01). These changes were reduced after silymarin treatment for 8 weeks (P < 0.05; P < 0.01). These findings suggest that treating diabetic rats with silymarin could decrease the levels of AGEs, and RAGE might reduce the possible complications caused by AGEs. It appears that silymarin exerts an AGE inhibitor property in the retina.

**Figure 4:**
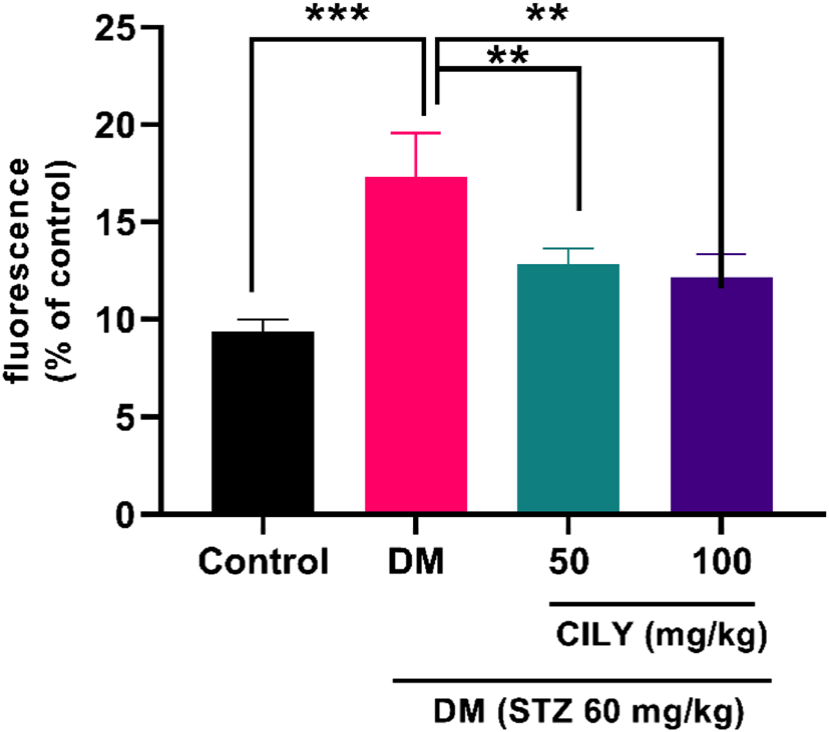
Silymarin attenuates ROS levels in the retina of diabetic rats. ROS levels were measured using 2′-7′-dichlorofluorescein-diacetate (DCFH-DA). Data are reported as mean ± SD. The differences between groups were compared using one-way ANOVA and Tukey’s post hoc test. Eight animals in each group were utilized for this experiment. ** and *** showing *p* < 0.01 and *p* < 0.001, respectively. DM: diabetes mellitus; SILY: silymarin.

In diabetes, the AGE-RAGE interaction could trigger several intracellular signaling pathways involved in the microvascular damage, including ROS overproduction^50,51^. In response to AGEs, elevated intracellular levels of ROS act as mediators of signal transduction pathways and subsequently caused cellular toxicity. It has been supported that AGEs stimulated retinal degeneration through ROS overproduction in both in vitro and animal models^52^. We indicated that the retinal ROS levels were dramatically higher in the diabetic rats than the healthy rats (Fig. 4; P < 0.001). Many in-vivo and in-vitro studies have recently been conducted based on the potential antioxidant role of silymarin^13,53^. The antioxidant activity of silymarin is probably due to its polyphenol and flavonoid constituents. Stolf et al. illustrated that the administration of silymarin controlled diabetic complications in the pancreas, kidneys, and liver of STZ-induced mice by alleviating the inflammatory responses and improving the antioxidant defenses^15^. In another experimental study, Goli and her co-workers elucidated that total oxidant and malondialdehyde in the diabetic rats treated with silymarin were dramatically reduced^54^. The positive effect of silymarin on redox control was also postulated in the current study, and our findings were in line with the above studies. In the present work, after 8 weeks of treatment with 50 mg/kg silymarin, the retinal levels of ROS were remarkably attenuated in the treated diabetic groups relative to the untreated diabetic group (Fig. 4; P < 0.01). Likewise, 100 mg/kg of silymarin treatment lowered retinal ROS levels in diabetic rats (Fig. 4; P < 0.01).

**Figure 5:**
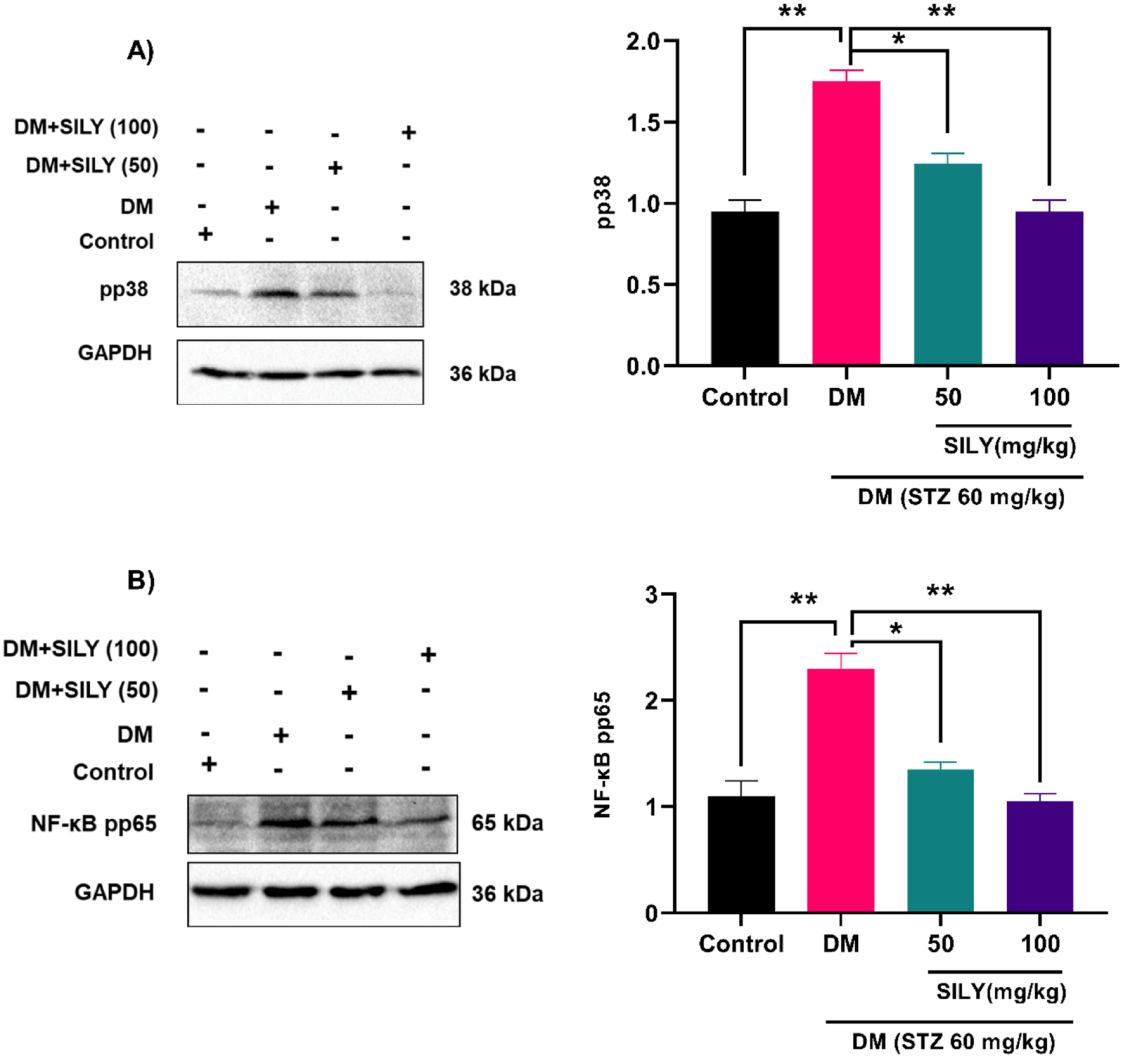
Silymarin attenuates (**A**) p38 MAP kinase and (**B**) NF-κB p65 phosphorylation in the retina of diabetic rats. Western blotting was applied to quantify p38 MAP kinase and NF-κB p65 phosphorylation. Data are reported as mean ± SD. The differences between groups were compared using one-way ANOVA and Tukey’s post hoc test. Eight animals in each group were utilized for this experiment. The original blots were presented in Supplementary Fig. S2A,B. * and ** showing *p* < 0.05 and *p* < 0.01, respectively. DM: diabetes mellitus; SILY: silymarin.

Moreover, ROS has been proved to activate MAP kinase signaling pathways in different cell types^55^. Shi et al. found that AGEs provoke apoptosis in human corneal epithelial cells through overproduction of ROS and activating the p38 MAP kinase signaling cascade^56,57^. In the present in vivo study, the phosphorylation of p38 MAP kinase was substantially raised in the diabetic animals relative to the healthy animals (P < 0.01) and was dramatically declined by silymarin treatment (Fig. 5A; P < 0.05; P < 0.05).

The p38 MAP kinase signaling pathway controls the activity of multiple transcription factors. Since NF-kB is extensively considered a major target of p38 MAP kinase in different tissues, we decided to peruse the involvement of this transcription factor in the pathological process of diabetic retinopathy. As illustrated in Fig. 5B, the phosphorylation of NF-κB p65 was greatly raised in the diabetic animals relative to the healthy animals (P < 0.01) and was dramatically attenuated by silymarin treatment (Fig. 5B; P < 0.05; P < 0.01). Hence, our findings are in line with prior studies that suggest increased NF-κB activity in the state of diabetic retinopathy. In a previous study, Wu et al. found that the action of NF-κB was enhanced in the retinas of rats with diabetic retinopathy^58^. In another study, Amin et al. reported that the administration of diabetic retinopathy rats with anti-RAGE led to suppression of NF-kB expression and inflammatory process in retinal tissue^59^. NF-κB may contribute to the progression of diabetic retinopathy by stimulating the expression of target genes, such as proinflammatory mediators, angiogenic agents, extracellular matrix proteins, and adhesion molecules^60,61^.

**Figure 6:**
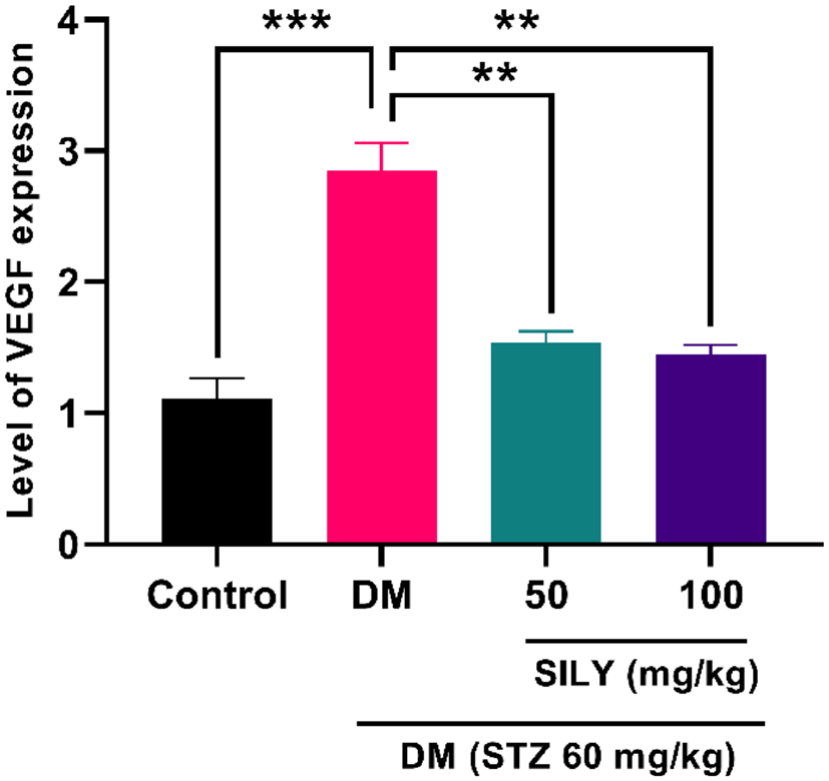
Silymarin attenuates VEGF levels in the retina of diabetic rats. Q-RT-PCR was applied to quantify mRNA. Data are reported as mean ± SD. The differences between groups were compared using one-way ANOVA and Tukey’s post hoc test. Eight animals in each group were utilized for this experiment. ** and *** showing *p* < 0.01 and *p* < 0.001, respectively. DM: diabetes mellitus; SILY: silymarin; VEGF: Vascular endothelial growth factor.

**Figure 7:**
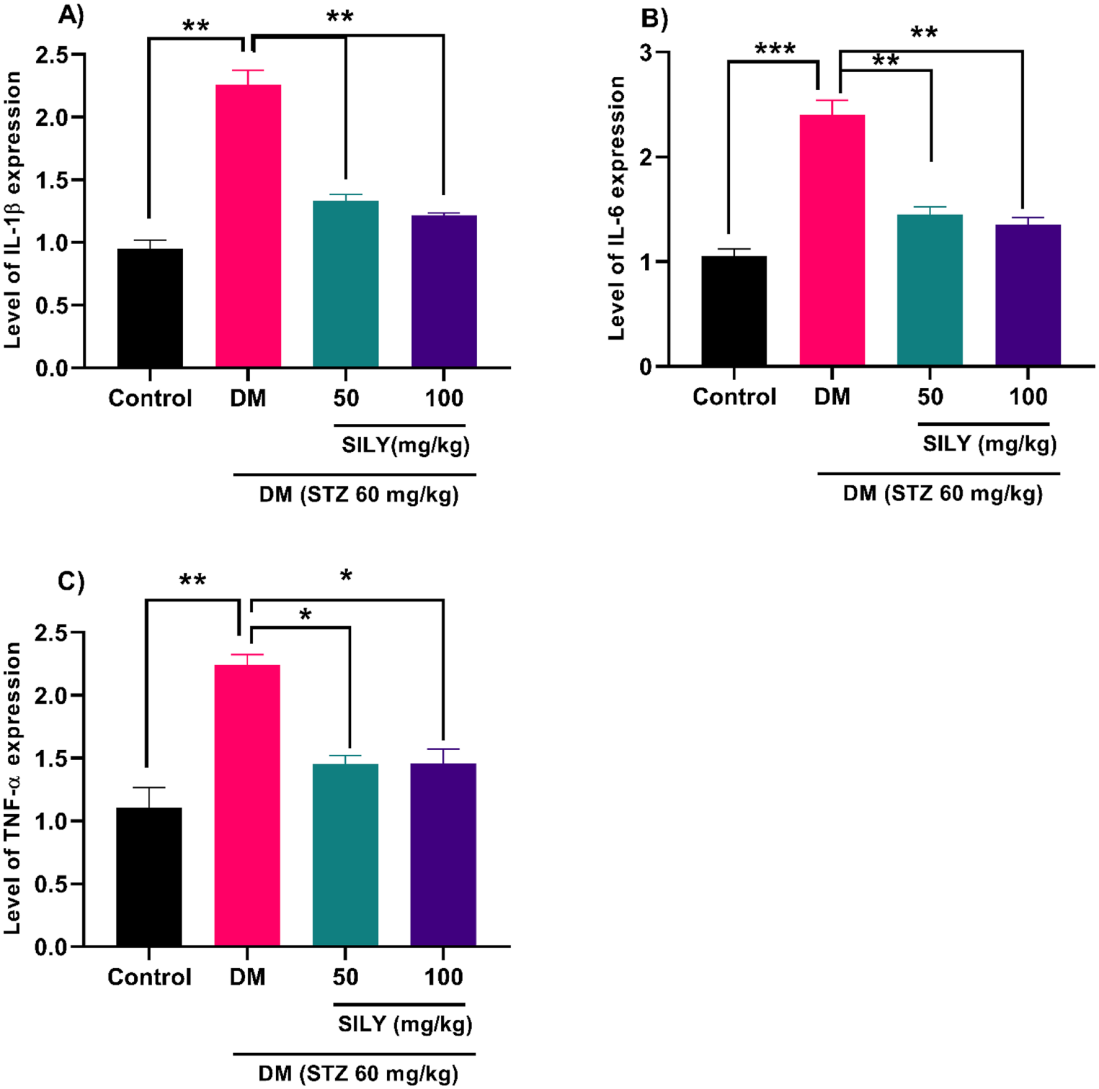
Silymarin attenuates (**A**) IL-1β, (**B**) IL-6, and (**C**) TNF-α mRNA levels in the retina of diabetic rats. Q-RT-PCR was applied to quantify mRNA levels. Data are reported as mean ± SD. The differences between groups were compared using one-way ANOVA and Tukey’s post hoc test. Eight animals in each group were utilized for this experiment. *, **, and *** showing *P* < 0.05, *p* < 0.01, and *p* < 0.001, respectively. DM: diabetes mellitus; SILY: silymarin.

Under physiological conditions, the angiogenesis process remains normal, but in pathological states such as diabetic retinopathy, the level of angiogenic genes such as VEGF elevates. There is a significant relationship between AGEs accumulation and VEGF expression, an angiogenic cytokine, in diabetic retinopathy^7^. Tao et al. provided evidence that demonstrated the expression of angiogenic genes was potentiated in the human retinal capillary endothelial cells following AGE treatment^8^. Numerous investigations have indicated that VEGF promoted vessel leakage and BRB breakdown, causing tissue edema^8,62^. In this condition, proinflammatory cytokines provoke the endothelium to overexpress adhesion molecules on the luminal surface of the endothelium, leading to leukocytes/monocytes recruitment and blood flow obstruction^63^. Due to the involvement of VEGF and inflammatory responses in the pathogenesis of diabetic retinopathy, particular attention is directed to pharmacological agents with anti-VEGF and anti-inflammatory effects for the reduction of issues in diabetic patients^9,64^. Many herbs' anti-inflammatory and antiangiogenic activities have been authenticated in previous studies. In previous research, Tzeng et al. revealed that Zerumbone isolated from Zingiber zerumbet attenuated hyperglycemia-induced inflammatory responses in STZ-stimulated diabetic rats. This flavonoid also ameliorated VEGF overexpression in the retinas of diabetic rats^26^. Recent studies suggest that administrating silybin to STZ-induced diabetic retinopathy rats suppressed angiogenesis, inflammation, and retinal vascular leukocytosis^39^. Here, diabetic rats revealed an obvious elevation in the retinal VEGF (Fig. 6; p < 0.001), IL-1β (Fig. 7A; P < 0.01), IL-6 (Fig. 7B; p < 0.001), TNF-α (Fig. 7C; P < 0.01) transcript levels compared to the healthy rats.

**Figure 8:**
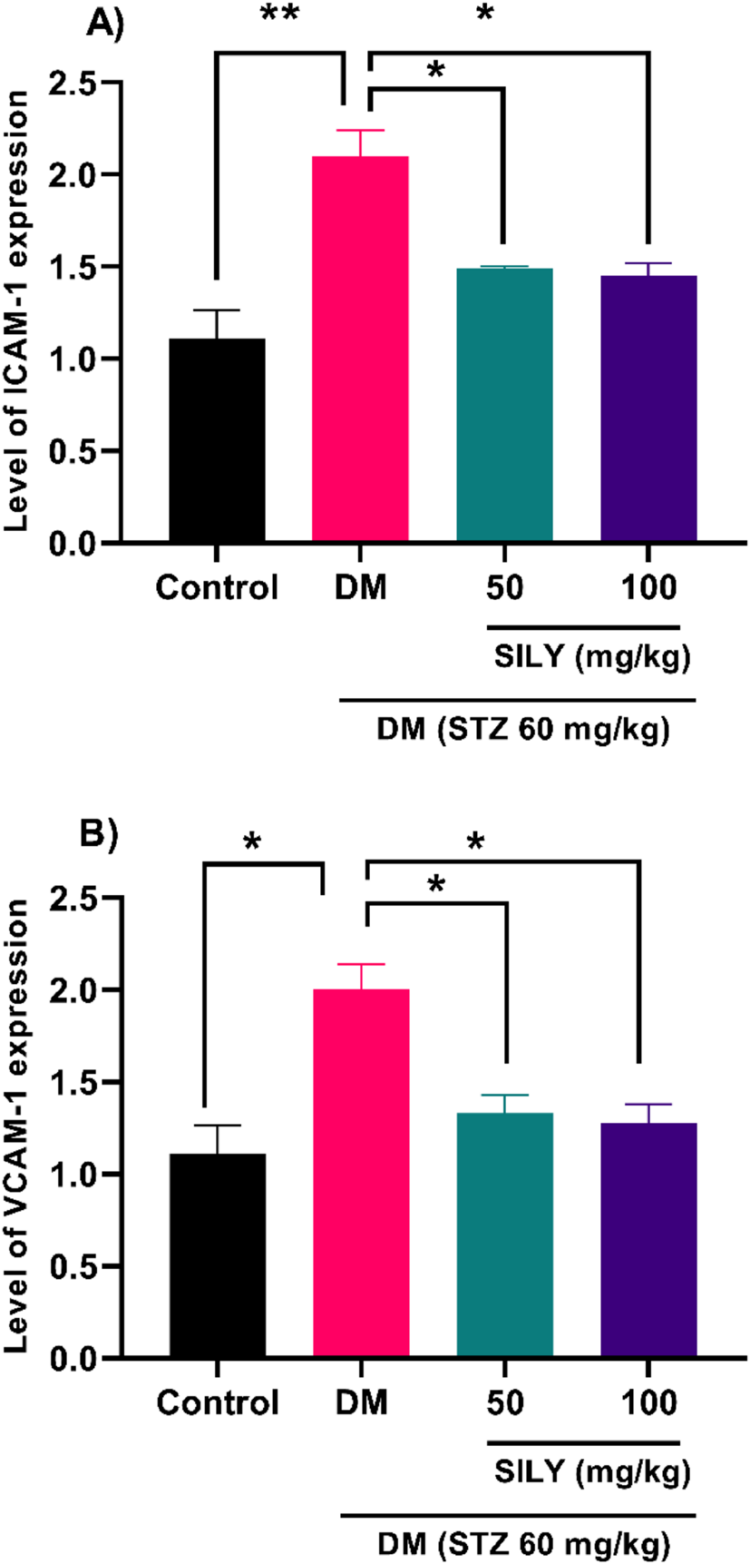
Silymarin attenuates (**A**) ICAM-1 and (**B**) VCAM-1 level in the retina of diabetic rats. Q-RT-PCR was applied to quantify mRNA levels. Data are reported as mean ± SD. The differences between groups were compared using one-way ANOVA and Tukey’s post hoc test. Eight animals in each group were utilized for this experiment. * and ** showing *P* < 0.05 and *p* < 0.01, respectively. DM: diabetes mellitus; SILY: silymarin, ICAM-1: Intercellular Adhesion Molecule 1, VCAM-1: vascular cell adhesion molecule 1.

We revealed that silymarin also alleviated the transcript levels of retinal VEGF in diabetic rats (Fig. 6; P < 0.01). The administration of silymarin led to a reduction in the transcriptional levels of IL-1β (Fig. 7A; P < 0.01), IL-6 (Fig. 7B; p < 0.01), TNF-α (Fig. 7C; P < 0.05) in STZ-induced diabetic rats. Therefore, silymarin can protect diabetic retinopathy through its anti-VEGF and anti-inflammatory properties. We also found a considerable enhancement in the transcript levels of ICAM-1 (Fig. 8A; P < 0.01) and VCAM-1 (Fig. 8B; P < 0.05) in the diabetic group relative to the healthy group. These changes were notably reduced in the silymarin-treated diabetic groups relative to the untreated diabetic group (P < 0.05).

**Figure 9:**
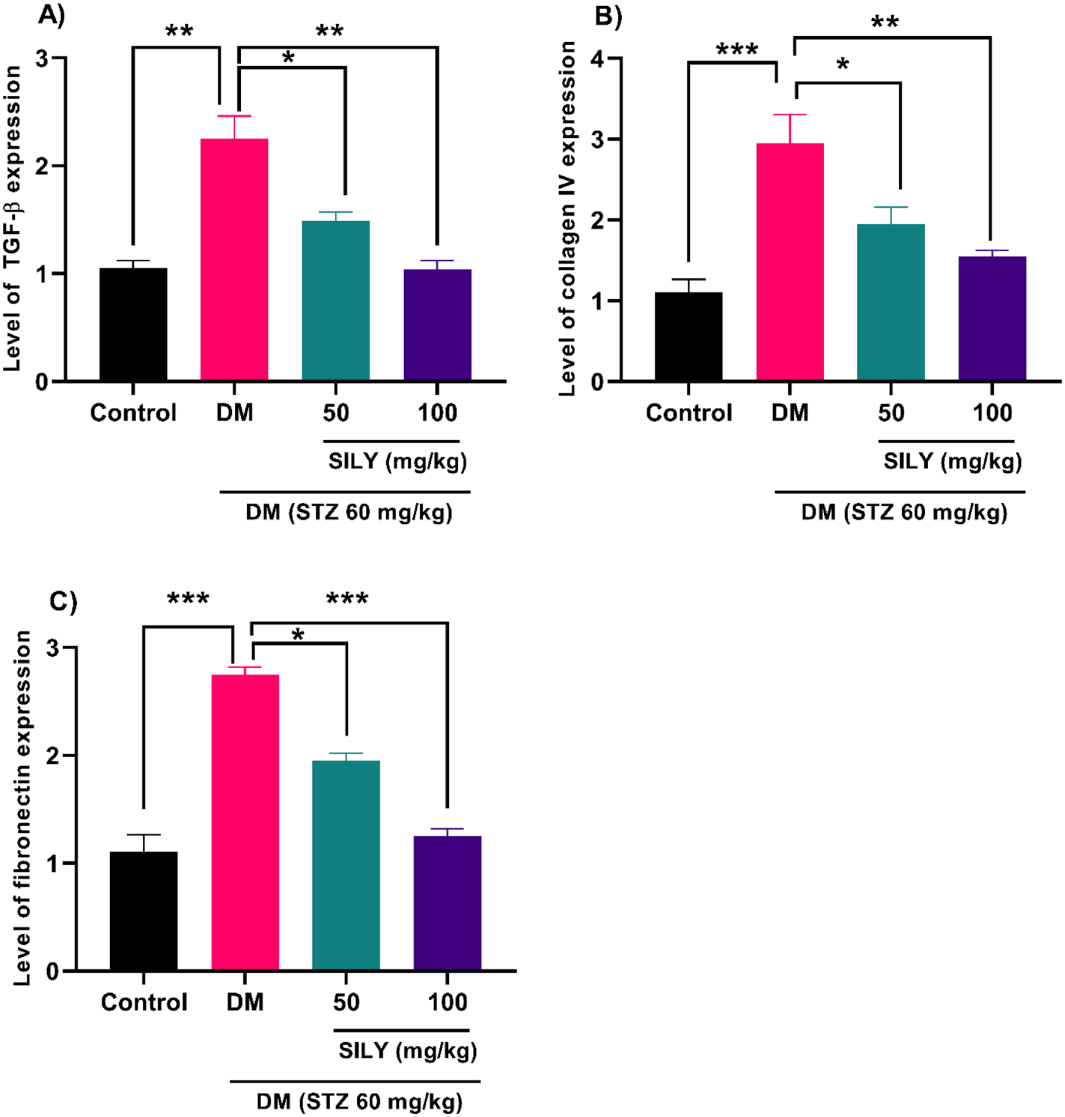
Silymarin attenuates (**A**) TGF-β, (**B**) collagen IV, and (**C**) fibronectin levels in the retina of diabetic rats. Q-RT-PCR was applied to quantify mRNA levels. Data are reported as mean ± SD. The differences between groups were compared using one-way ANOVA and Tukey’s post hoc test. Eight animals in each group were utilized for this experiment. *, **, and *** showing *P* < 0.05, *p* < 0.01, and *p* < 0.001, respectively. DM: diabetes mellitus; SILY: silymarin; TGF-β: transforming growth factor β.

Previous studies also disclosed that hyperglycemia provoked the expression of extracellular matrix molecules in retinal epithelial cells^65,66^. Qin et al. manifested that hyperglycemia promoted fibronectin and collagen IV expression in human retinal epithelial cells^67^. Increased deposition of extracellular matrix proteins is considered a hallmark of diabetic retinopathy^65^. Several studies documented that TGF-β is implicated in the pathogenesis of diabetic retinopathy via disrupting angiogenesis and BRB and increasing the accumulation of extracellular matrix proteins^67,68^. In our study, real-time PCR data revealed that TGF-β (Fig. 9A; P < 0.01), collagen IV (Fig. 9B; P < 0.001), and fibronectin (Fig. 9C; P < 0.001) transcript levels were notably augmented in the diabetic rats relative to the healthy rats. However, silymarin treatment for 8 weeks resulted in an obvious decrease in TGF-β, collagen IV, and fibronectin mRNA levels in the silymarin-treated diabetic rats relative to the untreated diabetic rats (P < 0.05, p < 0.01, p < 0.001).

Even though our study demonstrated that silymarin had a potential effect against retinal tissue damage in diabetes retinopathy, there are several limitations. Although our findings revealed that silymarin treatment could improve diabetic retinopathy, we cannot explain the silymarin efficacy relative to the standardized antidiabetic drugs. Also, we cannot specify which active compound of silymarin exhibits a protective effect in diabetic retinopathy.

## Conclusion

In conclusion, the current study's data revealed that regular administration of silymarin could ameliorate diabetic retinopathy in a diabetic animal model. Silymarin lowered serum glucose and reduced the levels of AGEs and RAGE in diabetic conditions. This resulted in inhibiting NF-κB and consequently attenuating inflammatory responses, adhesion molecules, and extracellular matrix proteins. We also revealed that these events depended on p38 MAPK activation, provoked by hyperglycemia in a redox-dependent pathway. We suggested that silymarin has favorable impacts on multiple targets in diabetic retinopathy. This finding may be useful for alternative therapies in diabetes clinical cases. However, further research is recommended to validate the present study.

## Supplementary Information

Supplementary Figures.

## Data Availability

The datasets generated during and/or analyzed during the current study are available from the corresponding author on reasonable request.
