# Supplementary material for: Silymarin reduces retinal microvascular damage in streptozotocin-induced diabetic rats
Source: Sci Rep. 2022 Sep 23;12:15872. doi: 10.1038/s41598-022-20297-2 (PMC9508129; doi:10.1038/s41598-022-20297-2)
Supplement: Supplementary file 1 — Supplementary Figures. [file 41598_2022_20297_MOESM1_ESM.docx]

**Supplementary document**

**Silymarin reduces retinal microvascular damage in streptozotocin-induced diabetic rats**

Rahman Karimi ^1^, Ali Bakhshi ^2^, Parisa Dayati ^3^, Omid Abazari ^2^, Maryamsadat Shahidi ^2^, Mohammadreza Savaee^2^, Ehsan Kafi ^2^, Mehdi Rahmanian ^4^, Seyed Morteza Naghib ^5*^

^1^ Department of Cell & Molecular Biology, Faculty of Biological Sciences, [Kharazmi University](https://scholar.google.com/citations?view_op=view_org&hl=en&org=6837845571097102989), Tehran, Iran

^2^ Department of Clinical Biochemistry, School of Medicine, Shahid Sadoughi University of Medical Sciences and Health Services, Yazd, Iran

^3^ Department of Clinical Biochemistry, Faculty of Medical Sciences, Tarbiat Modares University, Tehran, Iran

^4^ Biomaterials and Tissue Engineering Department, Breast Cancer Research Center, Motamed Cancer Institute, ACECR, Tehran 1517964311, Iran

^5^ Nanotechnology Department, School of Advanced Technologies, Iran University of Science and Technology (IUST), Tehran, Iran

* Correspondence: [naghib@iust.ac.ir](mailto:naghib@iust.ac.ir)

**Supplementary information:** Full blot images of western blot data

The membranes were used for several samples, so only the portions indicated in the boxes were included in the manuscript. The position of the bands was confirmed based on the ladder transferred on the PVDF membrane.

**Figure S1**


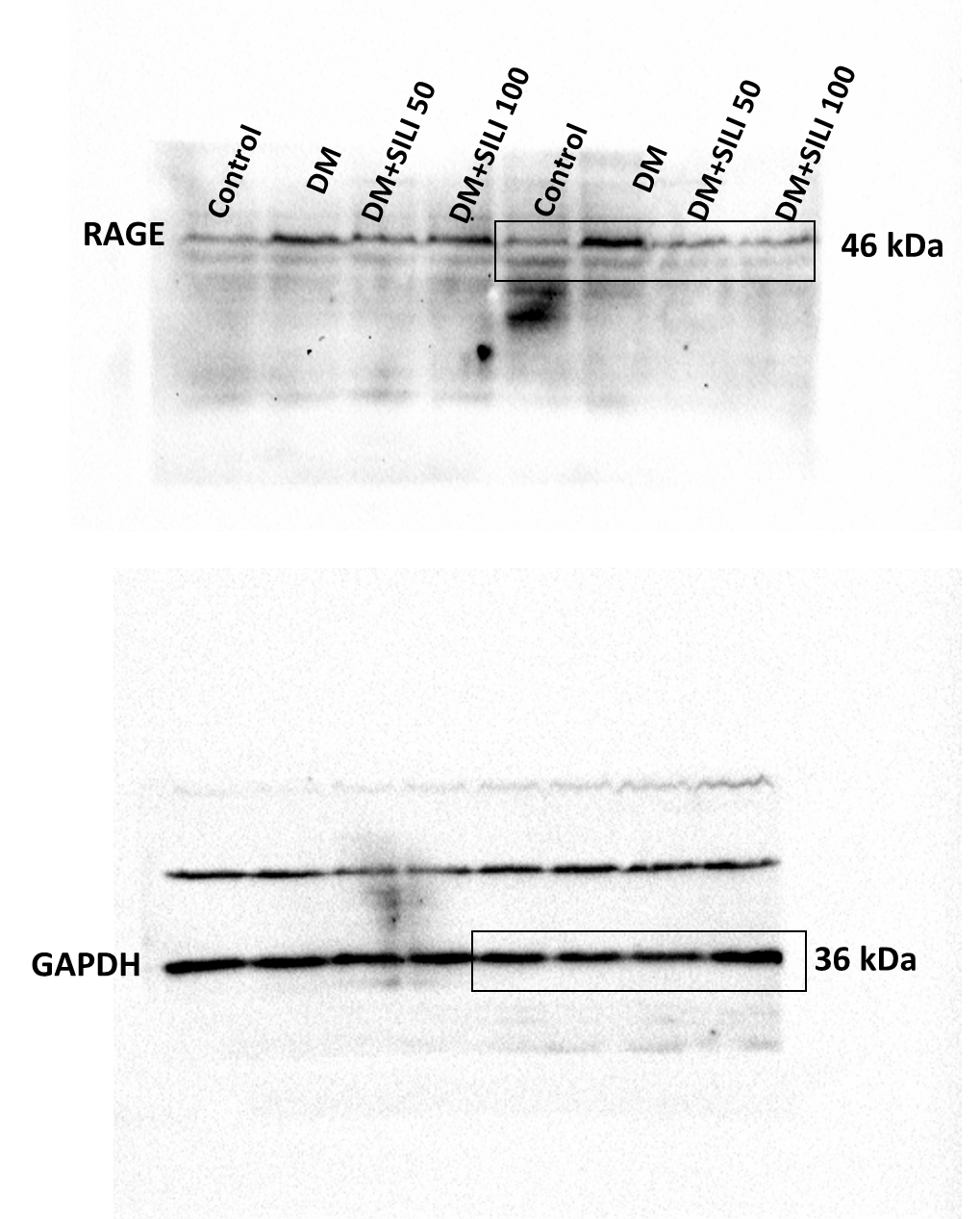


**Figure S2A**

**
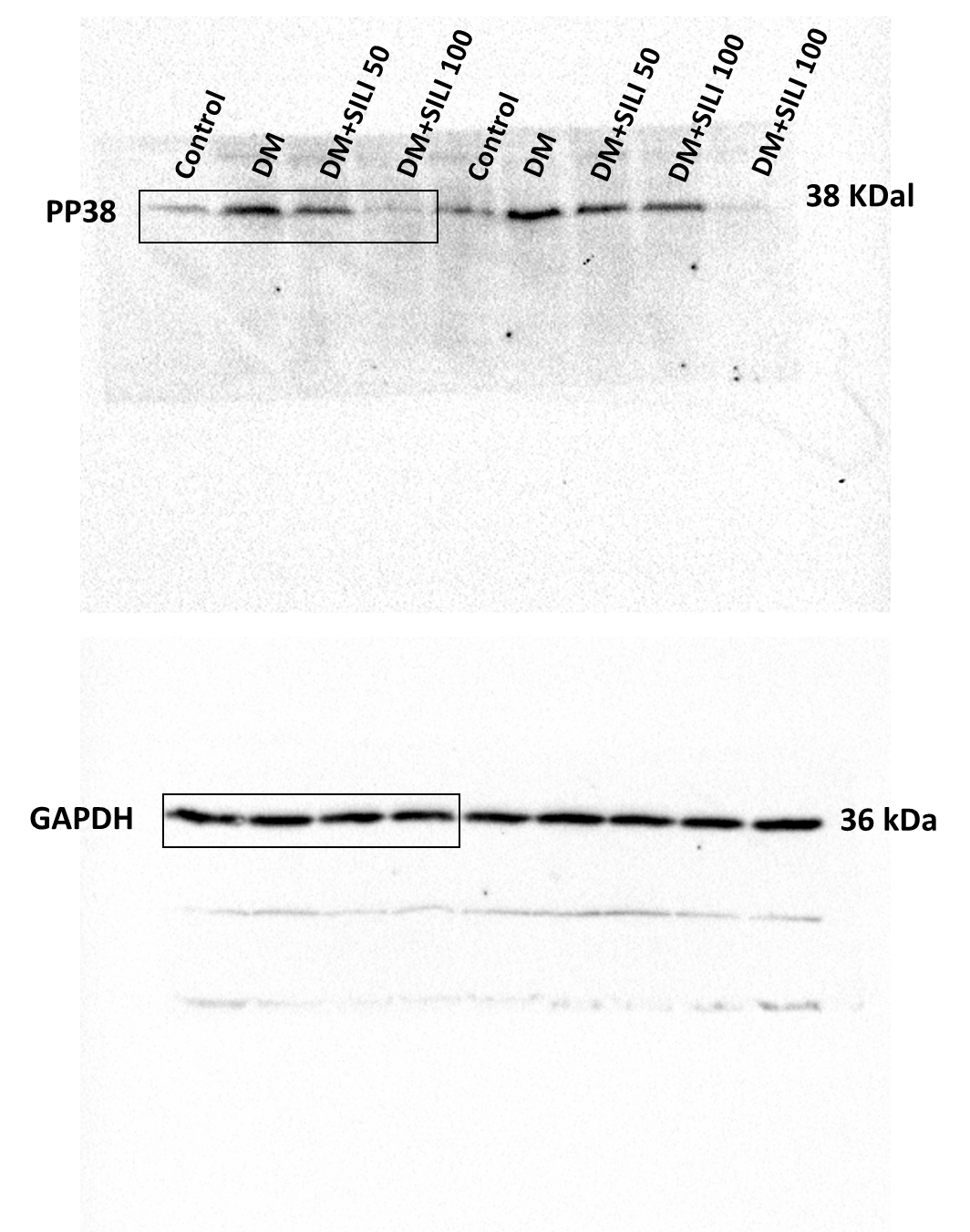
**

**Figure S2B**

**
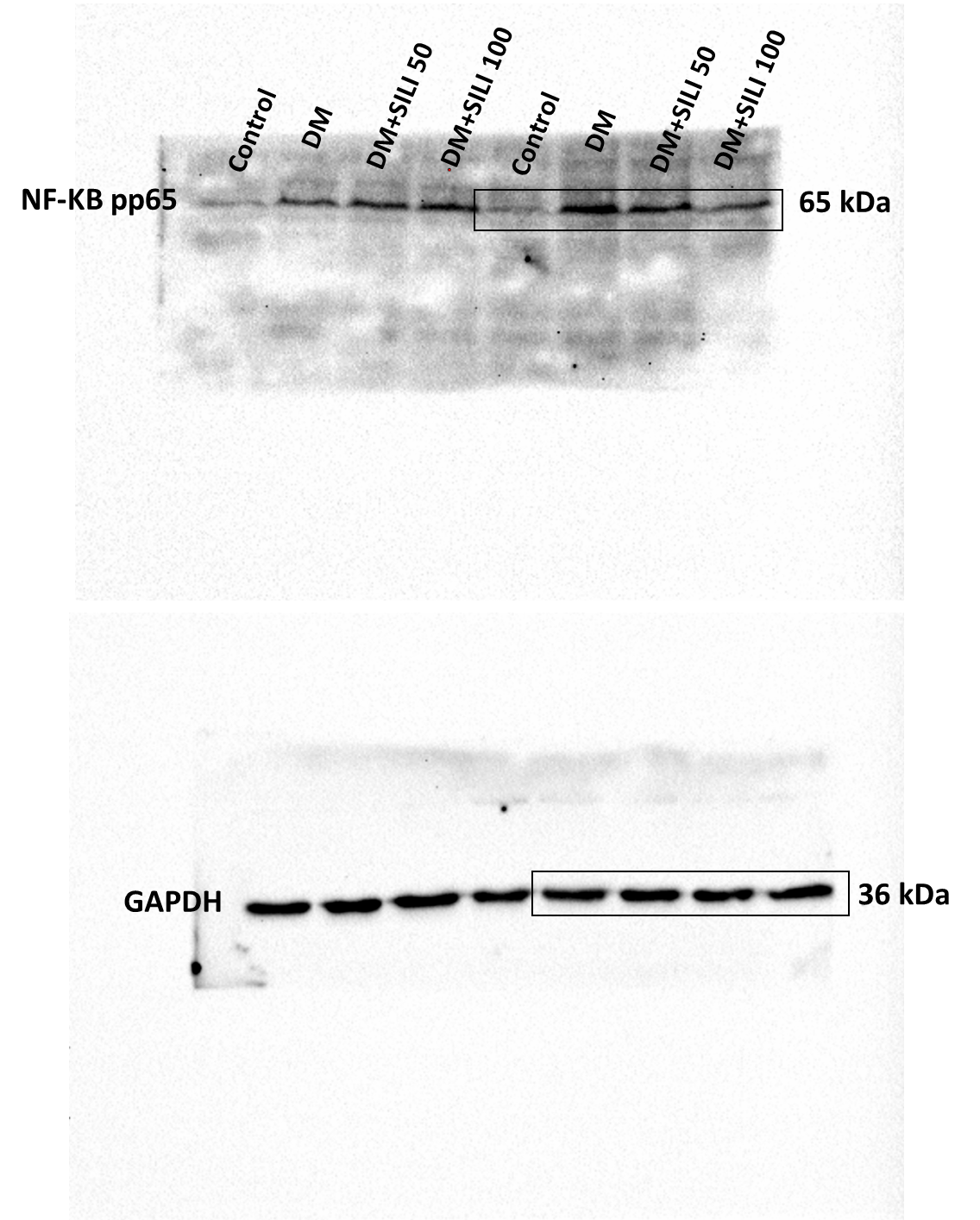
**
